# Variation and Selection in the Putative Sperm-Binding Region of ZP3 in Muroid Rodents: A Comparison between Cricetids and Murines

**DOI:** 10.3390/genes12091450

**Published:** 2021-09-20

**Authors:** Margarida Alexandra Duarte, Carlos Rodríguez Fernandes, Gerald Heckel, Maria da Luz Mathias, Cristiane Bastos-Silveira

**Affiliations:** 1 Champalimaud Centre for the Uknown, Champalimaud Research, Champalimaud Foundation, Avenida Brasília, 1400-038 Lisboa, Portugal; 2 Museu Nacional de História Natural e da Ciência, Departamento de Zoologia e Antropologia, Universidade de Lisboa, Rua da Escola Politécnica, 58, Lisboa, 1250-102 Lisboa, Portugal; 3 Departamento de Biologia Animal, Faculdade de Ciências da Universidade de Lisboa, Campo Grande, 1749-016 Lisboa, Portugal; mlmathias@fc.ul.pt; 4 Centro de Estudos de Ambiente e Mar, Faculdade de Ciências da Universidade de Lisboa, Campo Grande, 1749-016 Lisboa, Portugal; 5 cE3c-Centre for Ecology, Evolution and Environmental Changes, Departamento de Biologia Animal, Faculdade de Ciências, Universidade de Lisboa, 1749-016 Lisboa, Portugal; cafernandes@fc.ul.pt (C.R.F.); cbsilveira@gmail.com (C.B.-S.); 6 Faculdade de Psicologia, Universidade de Lisboa, Alameda da Universidade, 1649-013 Lisboa, Portugal; 7 Institute of Ecology and Evolution, University of Bern, Baltzerstrasse 6, CH-3012 Bern, Switzerland; gerald.heckel@iee.unibe.ch; 8 SIB Swiss Institute of Bioinformatics, Quartier Sorge-Batiment Amphipole, CH-1015 Lausanne, Switzerland

**Keywords:** zona pellucida glycoprotein 3, sperm receptor, female fertilization protein, positive selection, Cricetidae, Murinae

## Abstract

In mammals, the zona pellucida glycoprotein 3 (ZP3) is considered a primary sperm receptor of the oocyte and is hypothesized to be involved in reproductive isolation. We investigated patterns of diversity and selection in the putative sperm-binding region (pSBR) of mouse ZP3 across Cricetidae and Murinae, two hyperdiverse taxonomic groups within muroid rodents. In murines, the pSBR is fairly conserved, in particular the serine-rich stretch containing the glycosylation sites proposed as essential for sperm binding. In contrast, cricetid amino acid sequences of the pSBR were much more variable and the serine-rich motif, typical of murines, was generally substantially modified. Overall, our results suggest a general lack of species specificity of the pSBR across the two muroid families. We document statistical evidence of positive selection acting on exons 6 and 7 of *ZP3* and identified several amino acid sites that are likely targets of selection, with most positively selected sites falling within or adjacent to the pSBR.

## 1. Introduction

Gamete surface proteins can play an important role in reproductive isolation. They maintain species-specific barriers to fertilization and thus contribute to post-mating prezygotic isolation, and potentially to speciation [1,2,3]. In mammals, zona pellucida and sperm-head interacting proteins have co-evolved rapidly, presumably as a result of natural and sexual selection, leading to species-specific fertilization and genetic isolation [1,4,5]. This intersexual co-evolution is necessary to maintain gametic interaction and has led to amino acid differences between diverging populations [1,6]. Subsequently, gametic incompatibility may arise, promoting the differentiation of genomes and possibly ultimately the formation of new species.

One of the most studied reproductive proteins in mammals, both functionally and evolutionarily, is the zona pellucida glycoprotein 3 (ZP3), the sperm receptor of the oocyte and inducer of the acrosome reaction [7,8]. It consists of a polypeptide chain glycosylated with serine/threonine (O)-linked and asparagine (N)-linked oligosaccharides. ZP3 is a primary receptor during fertilization [9] because it binds directly to sperm, through its glycan chains, and inhibits further binding of sperm to the oocyte [10,11]. The putative sperm-binding region (pSBR), located in exon 7, exhibits considerable amino acid variation between species, which, together with modifications in the structure of the O-linked glycans, may enable a species-specific binding of sperm to the oocyte [7,8,12,13].

In house mice (*Mus musculus*), the best-studied system, sperm-oocyte interactions have been associated in particular to a serine (S) rich region (329–334), including the glycosylation sites S-332 and S-334, within the pSBR [14,15,16,17]. The classical model of sperm-oocyte binding proposes that gametic interactions occur via O-linked glycans attached to S-332 and S-334, and that after fertilization these residues are deglycosylated thereby preventing further sperm adhesion [14,17]. Studies using genetically modified mouse models have, however, challenged this classical view of sperm-oocyte binding and proposed alternative scenarios [18,19]. For example, it has been suggested that conserved O-linked glycosylation sites outside exon 7 and the pSBR are also exposed on the same 3D protein surface and constitute additional binding sites that may be involved in sperm-oocyte recognition [20,21], and/or sperm binding specificity may be based on the three-dimensional supramolecular structure of the zona pellucida, a matrix composed of ZP3 plus two additional proteins, ZP1 and ZP2 [22,23,24,25]. In fact, strong evidence has accumulated implicating ZP2, through a specific domain near its N-terminus, as a primary sperm receptor in mice [26,27]. It has very recently been suggested that the sperm-binding region may lie at the interface between the ZP2 and ZP3 subunits [28]. 

Although the molecular basis of sperm–oocyte binding remains incompletely understood, despite decades of investigation, and the exact role of the pSBR of ZP3 remains uncertain, it is clear that this glycoprotein, together with other zona pellucida and sperm head ligands, mediates sperm-oocyte binding, regardless of its specific molecular mechanism of action [2,29]. Moreover, species-specificity seems to be ensured both by the presence of a certain sperm receptor signature and by a particular glycosylation pattern of the glycoproteins of the zona pellucida, particularly ZP3 and ZP2 [19].

Several studies on the evolution of mammalian reproductive proteins have mainly consisted of comparing distantly related species, e.g., [30,31,32], but many more have focused on shorter evolutionary timescales, since fertilization mechanisms within species and among closely related taxa are more relevant to relate amino acid changes and reproductive isolation, e.g., [2,29,33,34,35,36,37,38]. This approach was tested in Cetartiodactyla, particularly in wild cattle [38] and cetaceans [37]. Both studies did not detect signatures of positive selection on ZP3 or evidence of its contribution to species specificity of sperm binding and prevention of cross-species fertilization. Data from rodent species, however, are contradictory. Turner and Hoekstra [34] documented positive selection acting on the pSBR of ZP3 in several deer mice (*Peromyscus*) species (Cricetidae, Neotominae), suggesting adaptive divergence within the genus. Analyses on Australian murine rodents (Muridae, Murinae) performed by Swann and colleagues [29] did not reach the same conclusions. 

Muroid rodents (Rodentia, Muroidea) are by far the largest extant mammalian superfamily, containing nearly one-third of all mammal species. In this study, we expanded the investigation of evolutionary patterns in the pSBR of ZP3 in its two most diverse families, Cricetidae and Muridae, by performing a comparative analysis of 93 species. Special focus is given to the speciose genus *Microtus* (meadow voles) (Cricetidae, Arvicolinae), an evolutionarily young group that started to radiate 1.2–2 million years ago [39]. It has given rise to 65 extant species [40], many of which are undergoing further diversification, e.g., [41,42,43,44].

## 2. Materials and Methods

### 2.1. Samples, DNA Extraction, Amplification and Sequencing

We examined 93 species of Cricetidae (N = 50) and Muridae (N = 43). Cricetid samples comprised 25 Arvicolinae (20 *Microtus* spp.), 17 Neotominae, four Cricetinae, two Tylomyinae and two Sigmodontinae species. All analyzed murid species were from the Murinae subfamily (Table S1). Tissue samples were provided by natural history museums and university research institutes (Table S1). Genomic DNA was extracted using standard protocols, with tissue digestion in a buffer containing sodium dodecyl sulfate (SDS) and proteinase K, followed by phenol-chloroform DNA extraction [45]. 

Exon 6, intron 6, and exon 7 of the *ZP3* gene were amplified using newly designed primers M-ZP3-F2 (5′-ATCACCTGTCATCTCAAAGTCA-3′) and M-ZP3-R1 (5′-CATGCCTGCGGTTTCTAGAAGC-3′). All polymerase chain reactions (PCR) contained 100 ng of genomic DNA, 0.3 mM of each primer, 1.25 U of GoTaq Flexi DNA Polymerase (Promega, Madison, WI, USA), 1x PCR buffer (Promega), 2.5 mM MgCl2, 0.1 μg of bovine serum albumin (BSA; New England Biolabs, Ipswich, United Kingdom), and 0.2 mM of each dNTP (Thermo Scientific, Waltham, MA, USA), and water up to a final volume of 25 μL. PCR amplifications were performed in a MyCycler thermal cycler (Bio-Rad Laboratories Inc., Hercules, CA, USA) and consisted of denaturation at 95 °C for 5 min, followed by 35 cycles of denaturation at 94 °C for 1 min, annealing at 58 °C for 1 min and extension at 72 °C for 1 min, and a final extension step at 72 °C for 10 min. The size of the PCR products was verified by electrophoresis in 1% agarose gels and comparison with GeneRuler™ 100 bp Plus DNA Ladder (Fermentas, Waltham, MA, USA). PCR products were purified with ExoI/FastAP (Fermentas). Sequencing in both directions, with the same primers used for the PCR reactions, was carried out by Macrogen Inc. (South Korea and the Netherlands) using an ABI Prism 3100 Genetic Analyzer (Applied Biosystems, Waltham, MA, USA).

Sequences were submitted to GenBank (accession numbers MT226280-MT226326; see Table S1 for details).

### 2.2. Sequence Analyses

Sequences were aligned using Sequencher 4.8 (Gene Codes Corporation) and BioEdit 7.2.5 [46]. We supplemented our sequence dataset with GenBank *ZP3* sequences of Arvicolinae, Neotominae, and Murinae taxa (Table S1). We included a species representing each of the murine genera analyzed.

In subsequent analyses, we focused on the coding regions of exons 6 and 7 because of their potential importance in ZP3 for the species specificity of sperm binding. Sequences were collapsed into unphased genotypes using the DNAcollapser tool in FaBox 1.5 [47]. Heterozygous positions in the larger intraspecific datasets (*Microtus lusitanicus* and *Microtus duodecimcostatus*) were phased using Phase 2.1.1 [48,49] as implemented in DNAsp 5.10.1 [50]. Five independent runs were conducted using default values, and after checking for concordance a final run with 10 times more iterations (1000 iterations and 1000 burn-in) was performed. Heterozygous positions of smaller intraspecific datasets were phased manually. DNA polymorphism parameters, such as the number of variable sites, number of parsimony-informative sites, number of non-synonymous sites, nucleotide diversity (π), and GC content were calculated in DnaSP. The translation of DNA sequences into amino acid sequences was performed with BioEdit. Amino acid sequence conservation and variation were visualized using the WebLogo application [51,52] via the SIB ExPASy Bioinformatics Resource Portal [53].

JModelTest 2.1.7 [54] was used to select the best-fitting model of nucleotide substitution (TVM+G, [55]) based on the Akaike information criterion (AIC) [56]. There were several species with gaps in the alignment of exon 7 sequences, and we wanted to include these indels in the phylogenetic analyses. Bayesian inference with MrBayes 3.1.2 [57,58] allows the incorporation of gaps coded as binary characters in a separate partition with a phylogenetic mixed model. Binary matrices were constructed with SeqState 1.4.1 [59], using two types of gap-coding: the simple indel coding (SIC, [60]) and modified complex indel coding (MCIC, [61]). Each Bayesian analysis consisted of two parallel Markov Chain Monte Carlo (MCMC) runs with four chains, one cold and three heated, for four million generations, with every 100th generation sampled. We determined convergence between the two runs when the average standard deviation of split frequencies was <0.01 [57]. The first 25% of trees were discarded as burn-in, and the remaining trees were used to construct a consensus tree and estimate Bayesian posterior probabilities. The consensus tree obtained was drawn using FigTree 1.3.1 [62]).

Since recombination may confound selection analyses [63,64,65], we tested for its presence using a set of methods implemented in RDP 4 [66]: RDP [67], BOOTSCAN [68,69], GENECONV [70], MAXCHI [71,72], CHIMAERA [72], SISCAN [73], and 3SEQ [74].

We tested for positive selection using the CodeML subroutine of PAML 4.8 [75,76]. Maximum likelihood estimates of ω (nonsynonymous (dN)/synonymous (dS) substitution ratio) across codons were inferred under seven models of variable ω among sites: M0 (one ω), M1a (nearly neutral, one ω, two classes of sites), M2a (positive selection, three classes of sites), M3 (discrete, three classes of sites); M7 (nearly neutral with β distribution approximating ω variation, 10 classes of sites), M8 (positive selection with β distribution approximating ω variation, 11 classes of sites) and M8a (ω distribution follows a mixture between a β distribution and a point mass at ω = 1, 11 classes of sites) [77,78,79,80,81,82,83]. The ω ratio is a sensitive measure of selective pressure, with positive selection inferred when ω > 1 [78,79]. 

Additionally, we used branch-site models that allow ω variation among amino acids in the protein and across branches on the phylogenetic tree in order to detect possible (episodic) positive selection affecting a few sites along particular lineages (foreground branches) [83,84,85,86]. In our case, this approach may allow us to detect positive selection affecting only a few amino acid residues in the analyzed fragment of ZP3 in specific lineages of the studied Muroidea. In fact, this strategy can be statistically more powerful than site-based tests, which average over all of the phylogeny [84]. The null (model = 2; NSsites = 2; ω = 1) and neutral M1a (model = 0; NSsites = 1; ω = 1) models were compared to the MA1 (model = 2; NSsites = 2; ω estimated), the alternative model in the branch-site test of positive selection [83,85]. The first comparison is a direct test for positive selection on the foreground lineages and therefore has been designated as the ‘branch-site test of positive selection’ [85], whereas the second test is also sensitive to relaxed purifying selection on the foreground branches [83,85]. Likelihood ratio tests (LRTs) of M0 vs. M3, M1a vs. M2a, M7 vs. M8, M8a vs. M8, null model vs. MA1 and M1a vs. MA1 were performed in order to search for evidence of positive selection [78,80,87]. Twice the log-likelihood difference between models (2∆l) was compared with a chi-square distribution with the number of degrees of freedom (dF) equal to the difference in the number of estimated parameters between the two models [80]. Positively selected sites under M2a, M3, M8, and MA1 were identified using the Naive Empirical Bayes (NEB) and the Bayes Empirical Bayes (BEB) approaches [83].

The M7-M8 test is the most powerful of the site models LRTs in PAML [82,88], but can also be biased towards false inference of adaptive evolution [38,88]. To further reduce the chances of falsely identifying sites as positively selected, we searched for signatures of positive selection using tests available in Datamonkey 2.0 [89,90,91], a web interface for the HyPhy package [92]. The tests carried out included individual site models that, unlike those available in CodeML, can incorporate synonymous substitution rate variation: SLAC (single likelihood ancestor counting, [93]), FEL (fixed effects likelihood, [93]), MEME (mixed effects model of evolution, [94]), and FUBAR (fast unconstrained Bayesian approximation, [95]). The other tests performed in Datamonkey were aBSREL (adaptive branch-site random effects likelihood, [96,97], an individual branch model that is an improved version of the branch-site models, and BUSTED (branch-site unrestricted statistical test for episodic diversification, [98]), a gene-wide test of episodic positive selection. All tests were performed with a significance threshold of 0.05.

## 3. Results

### 3.1. Genetic Variation and Phylogeny

**Figure 1:**
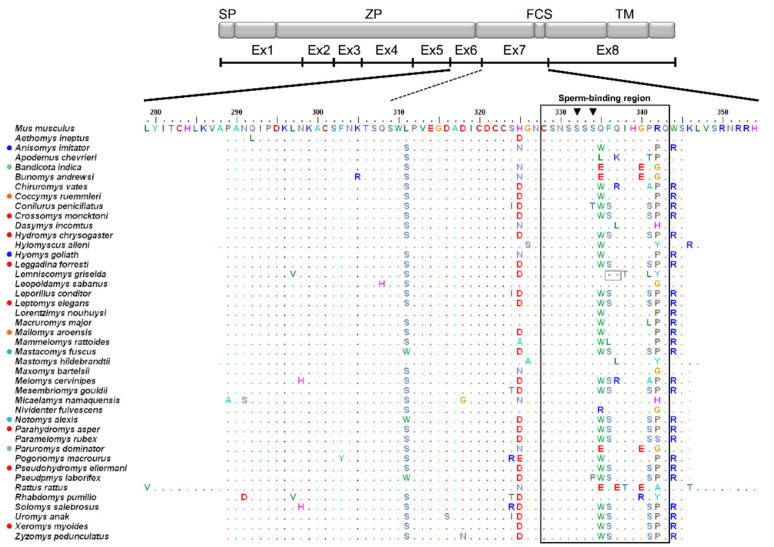
*ZP3* (zona pellucida glycoprotein 3) exons 6 and 7 amino acid sequence alignment of Murinae, with a schematic representation of the mouse protein and respective functional domains. Dots represent amino acids identical to the reference *Mus musculus* sequence and colored circles before species names denote shared haplotypes. The black outlined rectangle delimits the putative sperm-binding region according to [15]. Grey outlined squares highlight deletions relative to *M. musculus*. Black inverted triangles indicate glycosylation sites S-332 and S-334. SP = signal peptide, ZP = zona domain, FCS = furin cleavage site, TM = transmembrane domain. Ex1-Ex8: exons 1 to 8.

**Figure 2:**
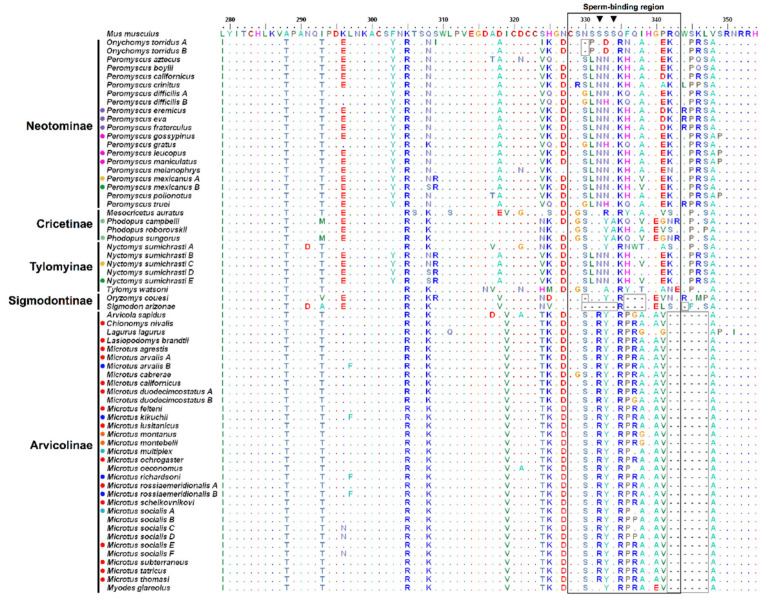
*ZP3* exons 6 and 7 amino acid sequence alignment of Cricetidae from the five extant subfamilies. Dots represent amino acids identical to the reference *Mus musculus* sequence and colored circles before species names denote shared haplotypes. The black outlined rectangle delimits the putative sperm-binding region according to [15]. Grey outlined squares highlight deletions relative to *M. musculus*. Black inverted triangles indicate glycosylation sites S-332 and S-334.

This study generated new sequences (N = 103, corresponding to 47 new haplotypes for exon 6 and 7 with GenBank accession numbers MT226280-MT226326) for 32 cricetid species. After the addition of previously published sequences of 18 cricetid and 43 murid species (Table S1), analysis of the resulting alignment revealed extensive length and sequence variation in *ZP3*, not only in intron 6 but also in exons 6 and 7, including the pSBR (Figure 1 and Figure 2 and Figure S1). The final data matrix containing only the coding regions was 228 base pairs (bp) long, corresponding to positions 835–1063 in the reference mouse *ZP3* gene. We did not observe length variation between the two alleles of an individual, and no evidence of recombination was found in the dataset by any of the detection methods employed. Twenty DNA sequences, four from GenBank and 16 newly produced herein, had heterozygous positions (16 at one position; three at two positions; one at three positions). The phased dataset contained a total of 78 variable sites, of which 63 were parsimony informative, and the GC content was 54.3%. The polymorphisms defined a total of 111 haplotypes, 40 in the murids and 71 in the cricetids (among the latter, 35 in the Arvicolinae, 23 in the Neotominae, four in the Cricetinae, six in the Tylomyinae, and three in the Sigmodontinae). No haplotypes were shared between families or subfamilies, but there was haplotype sharing among species of the same subfamily (Table S1). In total, there were nine haplotypes (12.7% of the cricetid haplotypes) shared between 14 Cricetidae species, almost all of them congeneric (*Microtus*), and two haplotypes (5% of the murine haplotypes) shared between five Murinae species.

No topological differences were observed between the trees from the two replicate MrBayes analyses for each of the three matrices derived from the alignment, excluding gaps or including them coded using SIC or MCIC (here we only present the phylogenetic tree derived using the SIC method, Figure S2). The only differences observed concern branch lengths of some lineages, which are explainable by the different treatment of indels by the three approaches used.

The phylogenetic reconstruction (Figure S2) grouped all species according to their family but yielded a topology within Cricetidae that is not congruent with the phylogeny of its subfamilies [99,100,101]. Indeed, while the tree obtained here showed essentially an unresolved polytomic relationship between the cricetid subfamilies (Figure S2), the established phylogeny supports two major clades: Arvicolinae + Cricetinae and Neotominae + Sigmodontinae + Tylomyinae [99,100,101]. The trees obtained from the matrices with gaps either excluded or coded using MCIC also had high support for most nodes (data not shown). The Arvicolinae and Sigmodontinae subfamilies were monophyletic, whereas the Cricetinae and Tylomyinae were not. Neotominae was also monophyletic, but this clade included as well haplotypes found in Tylomyinae taxa (Figure S2). The haplotype of the cricetine *Mesocricetus auratus* did not cluster with any subfamily and the haplotypes of the tylomyines *Tylomys watsoni* and *Nyctomys sumichrasti* grouped with the family Neotominae.

### 3.2. Amino Acid Variation

The translation of the DNA sequence of exons 6 and 7 yielded 74 amino acids (positions 279–354 according to the reference mouse ZP3 protein; [15]). Forty-five variable amino acid sites (60.8%) and 14 indel positions defined a total of 72 amino acid sequence types (Figure 1 and Figure 2, and Figure S1). Considerable length variation due to amino acid deletions, mainly in the pSBR (Figure 2, positions 328–343), was observed particularly in Arvicolinae and Sigmodontinae relative to murines (Figure 1). Compared to mouse ZP3, all arvicoline species lacked six amino acids at positions 342–347, and the two studied sigmodontines had amino acid deletions at positions 330 (also present in the neotomine *Onychomys torridus*) and 336–338 (Figure 2). Additional amino acid deletions were detected in *Sigmodon arizonae* (positions 331–334 and 344) (Figure 2). Therefore, the multiple amino acid deletions in the sigmodontines concern the serine-rich region at positions 329–334 and its immediate vicinity, whereas the six amino acid deletion in the arvicolines only involves the last two residues in the pSBR (Figure 2). In contrast to the subfamilies Arvicolinae, Neotominae, and Sigmodontinae, no deletion of amino acids relative to mouse ZP3 was found in Cricetinae and Tylomyinae (Figure 2). In turn, in the examined Murinae, amino acid deletions within the pSBR were only detected in *Lemniscomys griselda* (positions 336–337, Figure 1).

There were amino acid haplotypes shared between species of the same genus and even between genera of the same subfamily (Figure 1 and Figure 2 and Table S1). In cricetid genera represented by multiple species, 20 species of *Microtus* (Arvicolinae) had 11 amino acid haplotypes and 16 species of *Peromyscus* (Neotominae) showed 14 amino acid haplotypes (Figure 2 and Table S1). There were also cases of intraspecific polymorphism, with the presence of more than one amino acid haplotype, in arvicolines (Figure 2 and Table S1).

Considering only the sequences for the pSBR resulted in a decrease in the total number of amino acid haplotypes to 45, 24 for cricetids, and 21 for murines. Within the subfamilies of Cricetidae, there were eight haplotypes in arvicolines (four of them shared among different species), eight in neotomines (three shared between species), three in cricetines (one shared), three in tylomyines, and two in sigmodontines. There was only one case of shared haplotypes between cricetid species of different subfamilies, that between *P. mexicanus* (Neotominae) and *N. sumichrasti* (Tylomyinae). Among the haplotypes found in murines, five were shared by more than one species. In *Microtus* and *Peromyscus*, respectively, there were six (three shared between species) and seven (three shared between species) pSBR amino acid haplotypes.

In the data set for *Microtus*, the three pairs of well-accepted sister species, *M. duodecimcostatus*-*M. lusitanicus*, *M. felteni*-*M. thomasi* and *M. arvalis*-*M. rossiaemeridionalis* [102,103], all have areas of sympatry and share the same respective pSBR amino acid haplotype. In the *Peromyscus* data set, the sister species pairs consistently supported in the literature, *P. gossypinus*-*P. leucopus* [104,105,106] and *P. gratus*-*P. truei* [35,105,107], have both also areas of sympatry and also share the same respective pSBR amino acid haplotype. Finally, the cricetines *Phodopus campbelli* and *Phodopus sungorus* are sister species [108] with an area of sympatry, and also shared the same pSBR amino acid haplotype. 

The greatest variability in the analyzed ZP3 fragment occurred in the pSBR, in which only sites 328 and 339 were invariant in all species of cricetids and murines studied here (Figure 1 and Figure 2 and Figure S3), and adjacent amino acids (Figure 1 and Figure 2). Notably, almost all murine species examined, with the exception of *Conilurus penicillatus* and *Pseudomys laborifex* show conservation of the characteristic serine-asparagine-serine-serine-serine-serine sequence at positions 329–334 (SNSSSS) (Figure 1), whereas in no cricetid species this sequence is present and there is variability within each subfamily (Figure 2).

### 3.3. Selection Tests

The selection tests indicated that the analyzed *ZP3* sequences are under variable selective pressure among sites (Table 1 and Table S2). PAML LRTs rejected the null hypothesis site models M0, M1a, M7, and M8a in favor of the alternative M3, M2a, and M8 (*p* < 0.001 or *p* < 0.05) in tests with the full data set (Cricetidae + Murinae), Murinae only, and in *Microtus*, but only rejected the null hypothesis site model M0 in the analyses in Cricetidae and in *Peromyscus* (Table 1). The fact that for these last two data sets only the alternative model M3 was supported indicates that it was possible to detect variable selective pressure among sites but not positive selection [30,109]. For the different data sets, ω values < 1 in the supported site models indicate that most codons are under purifying selection (Table S2). For example, for the Cricetidae + Murinae data set the models M2a and M8 respectively estimated 65% and 88% of sites with ω < 1 and 9% and 12% of sites with ω > 1.

**Figure 3:**
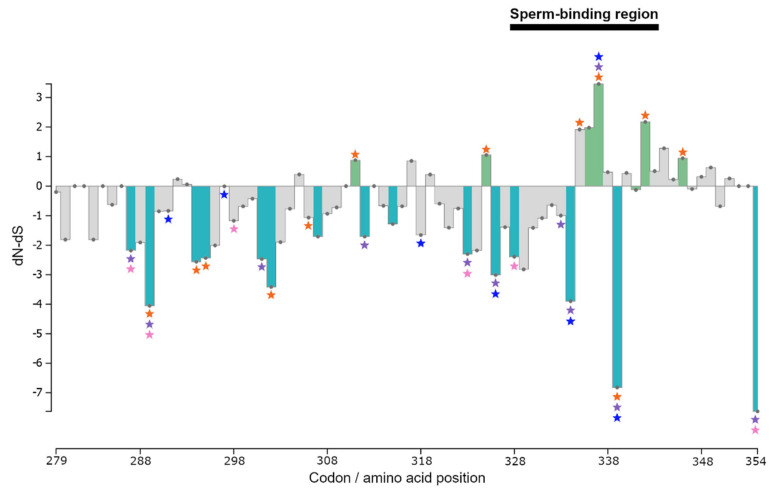
Distribution of amino acid sites under selection in exons 6 and 7 of *ZP3* as identified by PAML site models M2a, M3, and M8 and by HyPhy site tests SLAC, FEL, FUBAR, and MEME (*p* < 0.05). For the Cricetidae + Murinae data set, dN-dS columns corresponding to sites indicated as possibly being under positive selection by either all PAML models or all HyPhy tests are denoted in green, while columns of negatively selected sites in all HyPhy tests are shown in light blue. Grey columns correspond to sites that were not inferred to be under either positive or negative selection in all PAML and/or HyPhy tests. Coloured stars indicate sites selected only in particular data sets: purple = Cricetidae; orange = Murinae; blue = *Microtus*; and pink = *Peromyscus*. The normalized dN-dS per codon was calculated by SLAC.

Using the full data set (Cricetidae + Murinae), all selection methods applied identified both positively and negatively selected sites distributed throughout exons 6 and 7 of *ZP3* (Figure 3 and Table S2). With regard to the sites inferred to be under positive selection in the pSBR, site 337 was identified by all methods, site 336 was detected in all tests except SLAC, and sites 341 and 342 were indicated by all PAML site models (Figure 3 and Table S2). Yet another site in the pBSR, 335, was inferred as positively selected by all PAML site models on the Murinae data set (Figure 3 and Table S2). Outside the pSBR but still in its immediate vicinity, amino acids 311, 325, and 346 were determined to be under positive selection by all PAML site models in analyses of both the full data set and the murine dataset (Figure 3 and Table S2).

While in *Microtus*, in addition to site 337, residue 297 was indicated to be under positive selection by all PAML site models, in *Peromyscus* no consistent evidence of positive selection was found (Table S2). Overall, across analyses and data sets, most sites identified as positively selected fall within or adjacent to the pSBR (Figure 3). Fifteen sites were identified to be under purifying selection at p-value threshold 0.05 by HyPhy tests FEL, FUBAR, and SLAC on the Cricetidae + Murinae data set (Table S2). These included the serine-rich site 334 and the two invariant sites in the pSBR (C-328 and H-339) (Figure 3).

The PAML branch-site comparisons of the null model vs. MA1 and M1a vs. MA1 revealed variable selective pressure, depending on the family/subfamily set as the foreground branch (Table 1 and Table S2). The null hypothesis of no positive selection was rejected (*p* < 0.001) for both Murinae and Cricetidae, and for two cricetid subfamilies, the Arvicolinae and Tylomyinae. The MA1 model identified several sites, all outside the pSBR, in Murinae (315 and 322), Cricetidae (287, 307 and 311), Arvicolinae (287), and Tylomyinae (307 and 311) (Table S2) as positively selected. With the exception of site 311, all others were invariant across the entire data set and, thus, likely false positives [93,110].

Sampling and stochastic errors, model misspecification, and assumption violations, and testing of multiple foreground lineages, can lead to false positives [83,85,86,97,111,112,113]. For instance, it has been noted that the branch-site models in PAML may be sensitive to small sequences [29]. As noted by Zhang and colleagues [85], identifying sites under positive selection, especially in the case of episodic selection, is intrinsically more difficult than testing whether such sites exist, but it is still useful to be able to detect positive selection acting on a region. Importantly, site 311 was also suggested to be influenced by positive selection in all PAML site model tests with the full data set (Table S2). The HyPhy tests aBSREL and BUSTED found no evidence of episodic diversifying selection across the *ZP3* phylogeny.

## 4. Discussion

We investigated patterns of variation and natural selection at the pSBR and adjacent exonic sequences of the reproductive protein ZP3 in species of Cricetidae and Murinae. We found that murine pSBR is fairly conserved, in particular the serine-rich stretch containing the glycosylation sites, which has been proposed as essential for sperm binding, being invariant in 41 of the 43 murine genera examined. In contrast, the amino acid sequence of the pSBR was much more variable in cricetids, and the serine-rich motif typical of murines was generally substantially modified, implying that in Cricetidae the ZP3-mediated sperm binding does not follow the classical model proposed for the mouse. 

The virtual lack of sequence variation in the serine-rich region at positions 329–334 and the relatively high conservation of the entire pSBR in the murine species studied (Figure 1 and Figure S3) suggests a functional conservation of this region in murines. According to [29,36], the reduced intergeneric variability in the murine pSBR seems to indicate a limited role for this region in species-specific sperm-ZP binding, a scenario supported by our results as well.

In our study, of the six sites (311, 325, 335, 337, 342, and 346) that were consistently detected in different selection tests as potentially under positive selection in Murinae, sites 325 [29,30] and 311, 337 and 342 [30] have also been constantly strongly supported as positively selected in previous analyses in murines. Two of these sites (337 and 342) were also identified in a study of closely related species of the mouse genus *Mus* [33]. However, when this analysis was repeated without including outgroup sequences, no evidence for positive selection was found [34]. This case illustrates the advantages of the approach followed in our study. In addition to using multiple different positive selection tests, as recommended by many authors to increase the robustness of the results, e.g., [80,88,109,114], we conducted analyses at various phylogenetic levels using different hierarchical subsets of the full data set. 

With regard to Cricetidae, the pSBR and even the serine-rich region were remarkably variable (Figure 2, Figures S1 and S3). In fact, the serine-rich motif SNSSSS typical of murines was generally substantially modified in all cricetid subfamilies (Figure 2 and Figure S3). In particular, most cricetids do not have a serine at position 332, but on the other hand, they have a serine at position 330 (Figure 2 and Figure S3), which is apparently fixed for asparagine in Murinae (Figure 1). 

Our survey of the pSBR of ZP3 across all the subfamilies of the Cricetidae indicates that in this family the ZP3-mediated sperm-oocyte binding does not follow the classical model proposed for the mouse. In fact, not even all of the murines examined were conserved for serine at position 334 (Figure 1). According to the classical model suggested for the mouse, this would affect gamete interaction since S-332 and S-334 are hypothesized to carry O-linked glycans that are essential for sperm-oocyte binding. Hence, even within murines, there may be alternative mechanisms and other anchor amino acids crucial for gamete recognition.

The role attributed to ZP3 with regard to a species-specific function in gamete recognition may involve regions of the protein other than those encoded by exons 6 and 7. It has been proposed that sperm binds to ZP3 by interacting with O-linked glycans not linked to S-332 and S-334 [115] or with N-linked glycans and accessible protein regions located within the C-terminal domain of ZP3 [116]. In particular, it has been suggested that two conserved O-linked glycosylation sites (residues T-155 and T-162/S-164/S-165) shared by mouse and human ZP3, and which are exposed on the same 3D protein surface as the pSBR in exon 7, may be the actual attachment sites of the sperm-binding glycans [20,21]. Moreover, more recently, significant experimental evidence has indicated that the sperm-binding region of the zona pellucida may reside in ZP2 [26,27], and lately, it has even been proposed that it might lie at the interface between the ZP2 and ZP3 subunits [28]. Therefore, uncertainty and debate on this issue remain high [19,117].

**Figure 4:**
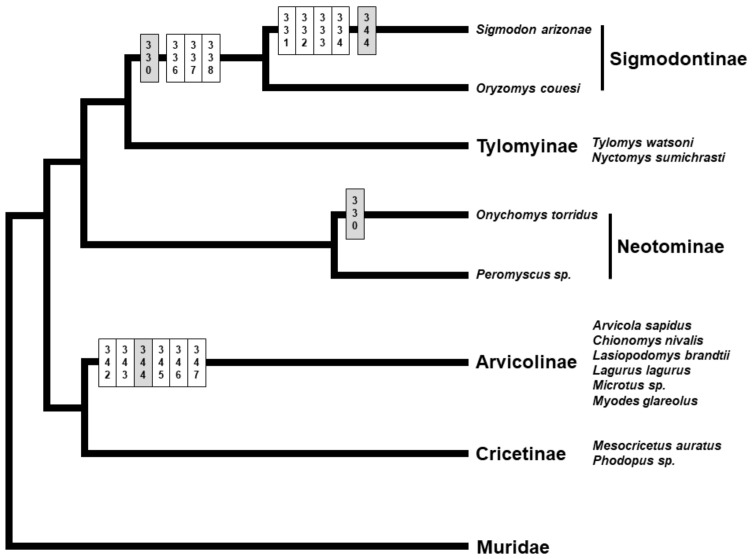
Schematic representation of the phylogenetic tree for the cricetid subfamilies (based on [99,100,101]), with the amino acid deletions observed in the putative sperm-binding region and its immediate vicinity in the ZP3 of different lineages indicated on the respective branches of the tree. Position numbers are according to the mouse reference sequence for ZP3. Sites in white are amino acid deletions unique to a lineage, while sites in grey are amino acid deletions shared between cricetid subfamilies.

The most extreme cases of divergence in the serine-rich region, due to multiple amino acid deletions, were detected in arvicolines and sigmodontines (Figure 2). Given the established phylogeny for the cricetid subfamilies [99,100,101], supporting the two clades Arvicolinae + Cricetinae and Neotominae + Sigmodontinae + Tylomyinae, the observed deletions do not seem to be associated with the evolutionary relationships among subfamilies, and the few deletions shared between different subfamilies appear to be clear cases of convergence (Figure 4).

We observed intraspecific amino acid variation in several species of *Microtus* and other cricetid genera (Table S1), similar to previous reports for *Peromyscus* [34,35]. Moreover, the inter-specific and -generic sharing of amino acid haplotypes may not only represent shared ancestral polymorphism but may also be due to convergence as a by-product of (balancing) selection maintaining divergent alleles within species [2,35].

Regarding a possible general involvement of the pSBR in the species specificity of fertilization in cricetids, this is seemingly contradicted by the extensive haplotype sharing between congeners and even among different genera (Figure 2). The fact that sister, or closely related species with sympatric areas of distribution, share pSBR amino acid haplotypes has been considered evidence of lack of selection for pSBR divergence to prevent or reduce hybridization [29,30,35]. However, this conclusion is only valid, and relevant, if the analyzed sister or closely related species were not sampled from allopatric populations. In this study, data from *Peromyscus* sister taxa *P. gossypinus* and *P. leucopus* [34] and *P. gratus* and *P. truei* [35] were, in both cases, from allopatric populations. Regarding *Microtus*, in the three pairs of sister species sharing haplotypes (*M. duodecimcostatus*-*M. lusitanicus*, *M. felteni*-*M. thomasi*, and *M. arvalis*-*M. rossiaemeridionalis*), only for the first pair the data were from sympatric locations. Genetic data from *M. duodecimcostatus* and *M. lusitanicus* revealed historical introgression of mitochondrial DNA, but low gene flow given the clear differences in nuclear DNA at the sympatry zone [42]. The factors maintaining the genetic integrity of the two taxa in the sympatric region remain unknown. However, this study and recent data from crossbreeding of sympatric individuals [118] indicate the development of prezygotic and postzygotic barriers but not gametic isolation.

## 5. Conclusions

In conclusion, the results of the present study indicate a general lack of species specificity of the pSBR in muroid rodents. However, our data are consistent with hypotheses and models that describe multiple distinct binding sites in sperm-oocyte recognition. Thus, we suggest that future studies should focus on the complete ZP3 and ZP2 proteins, and looking for signatures of coevolution, also with sperm head proteins, in order to compare and evaluate different proposed sperm-binding regions. Moreover, to clarify their potential role in the development and/or maintenance of reproductive isolation and speciation, efforts should be made to investigate closely related species in areas of sympatric distribution.

## Figures and Tables

**Table 1:** Results of the likelihood ratio tests (LRT) considering site- and branch-site models implemented by PAML on exon 6 and 7 of the *ZP3* gene of the analyzed.

Type	LRT	2∆l	d.f.	*p* Value
Site-models: Cricetidae + Murinae	M0 vs. M3	273.646	4	<0.001
	M1a vs. M2a	37.492	2	<0.001
	M7 vs. M8	42.397	2	<0.001
	M8a vs. M8	35.522	1	<0.001
Site-models: Cricetidae	M0 vs. M3	139.759	4	<0.001
	M1a vs. M2a	0.726	2	0.696
	M7 vs. M8	4.4671	2	0.107
	M8a vs. M8	3.490	1	0.062
Site-models: Murinae	M0 vs. M3	102.930	4	<0.001
	M1a vs. M2a	16.674	2	<0.001
	M7 vs. M8	21.164	2	<0.001
	M8a vs. M8	18.286	1	<0.001
Site-models: *Microtus*	M0 vs. M3	60.304	4	<0.001
	M1a vs. M2a	8.622	2	0.013
	M7 vs. M8	8.350	2	0.015
	M8a vs. M8	8.232	1	0.004
Site-models: *Peromyscus*	M0 vs. M3	15.034	4	0.004
	M1a vs. M2a	0.000	2	1.000
	M7 vs. M8	0.025	2	0.988
	M8a vs. M8	0.022	1	0.883
Branch-site models: Murinae	null vs. MA1	0.796	2	<0.001
	M1a vs. MA1	1.420	2	<0.001
Branch-site models: Cricetidae	null vs. MA1	1.077	2	<0.001
	M1a vs. MA1	12.208	2	<0.001
Branch-site models: Arvicolinae	null vs. MA1	6.280	2	<0.001
	M1a vs. MA1	6.560	2	<0.001
Branch-site models: Cricetinae	null vs. MA1	0.000	2	1.000
	M1a vs. MA1	0.927	2	0.629
Branch-site models: Neotominae	null vs. MA1	0.000	2	1.000
	M1a vs. MA1	0.000	2	1.000
Branch-site models: Sigmodontinae	null vs. MA1	0.000	2	1.000
	M1a vs. MA1	0.004	2	0.998
Branch-site models: Tylomyinae	null vs. MA1	0.087	2	<0.001
	M1a vs. MA1	0.242	2	0.242

## Data Availability

The data presented in this study are openly available in GenBank in accession numbers MT226280-MT226326. See Table S1 for details.

## Supplementary Materials

The following are available online at https://www.mdpi.com/article/10.3390/genes12091450/s1, Figure S1: *ZP3* exons 6 and 7 amino acid sequence alignment of Cricetidae, with a schematic representation of the mouse protein and respective functional domains, Figure S2: Bayesian inference phylogenetic tree obtained for exons 6 and 7 of *ZP3* of the studied muroid rodents, with indels coded using the SIC method, Figure S3: Amino acid sequence logos showing patterns of conservation and variation in the putative sperm-binding region of ZP3, for different data sets, Table S1: List of species examined in this study, Table S2: Detailed results of selection tests.
