# Supplementary material for: Variation and Selection in the Putative Sperm-Binding Region of ZP3 in Muroid Rodents: A Comparison between Cricetids and Murines
Source: Genes (Basel). 2021 Sep 20;12(9):1450. doi: 10.3390/genes12091450 (PMC8469249; doi:10.3390/genes12091450)
Supplement: Supplementary file 1 [file genes-12-01450-s001.zip › genes-1326298-supplementary.pdf]

Figure S1 *ZP3* exons 6 and 7 amino acid sequence alignment of Cricetidae, with a schematic representation of the mouse protein and respective functional domains. Dots represent amino acids identical to a cricetid reference sequence, *Peromyscus aztecus*. The black outlined rectangle delimits the putative sperm-binding region according to [15]. Grey outlined squares highlight deletions relative to *P. aztecus*. Black inverted triangles indicate glycosylation sites S-332 and S-334. SP = signal peptide, ZP = zona domain, FCS = furin cleavage site, TM = transmembrane domain. Ex1-Ex8: exons 1 to 8.

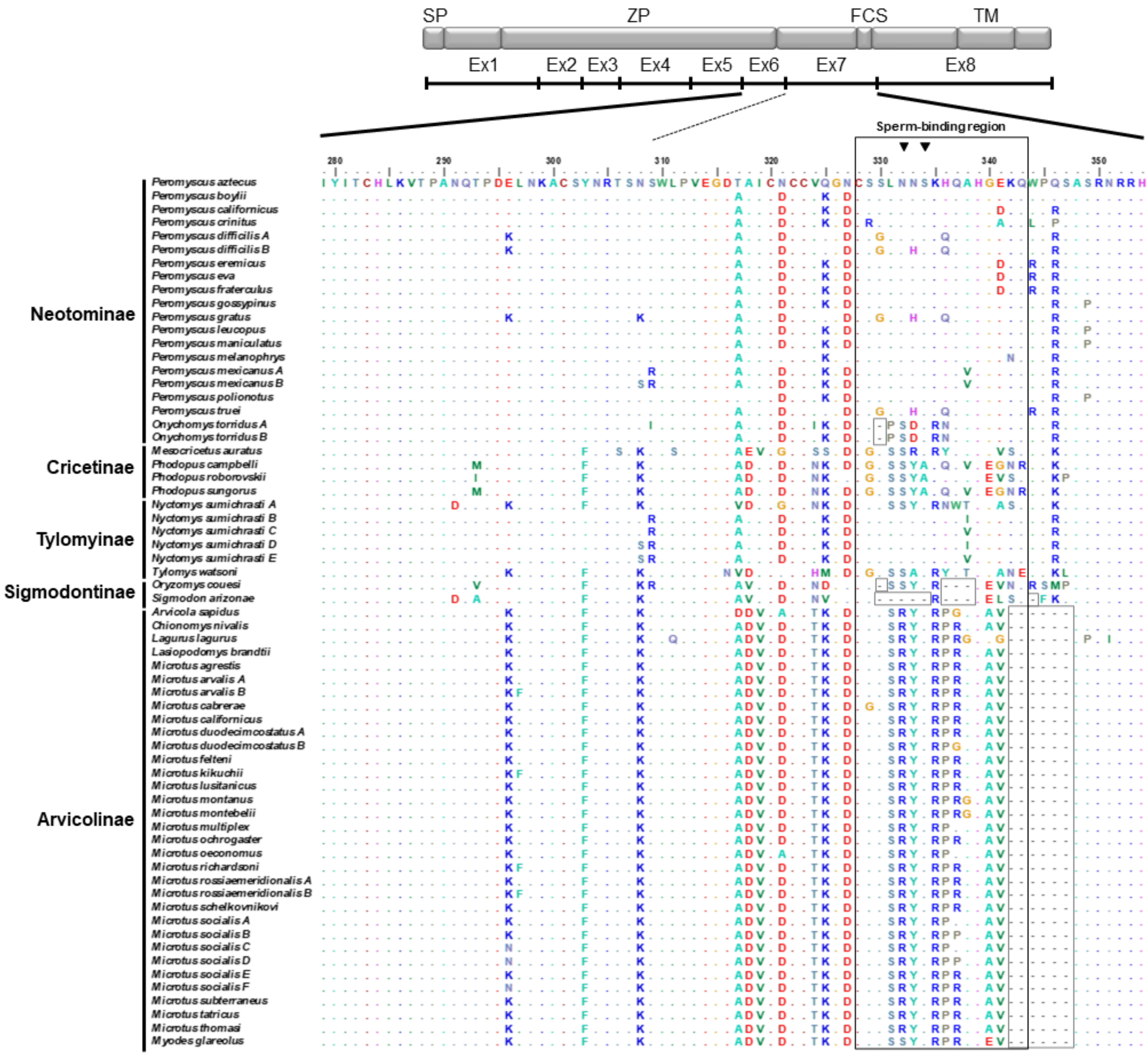

Figure S2 Bayesian inference phylogenetic tree obtained for exons 6 and 7 of *ZP3* of the studied muroid rodents, with indels coded using the SIC method. Shown are posterior probabilities for cricetid clades that were greater than 0.5. Clades and haplotypes of Cricetinae and Tylomyinae are respectively indicated by black and white circles because these two cricetid subfamilies were not monophyletic.

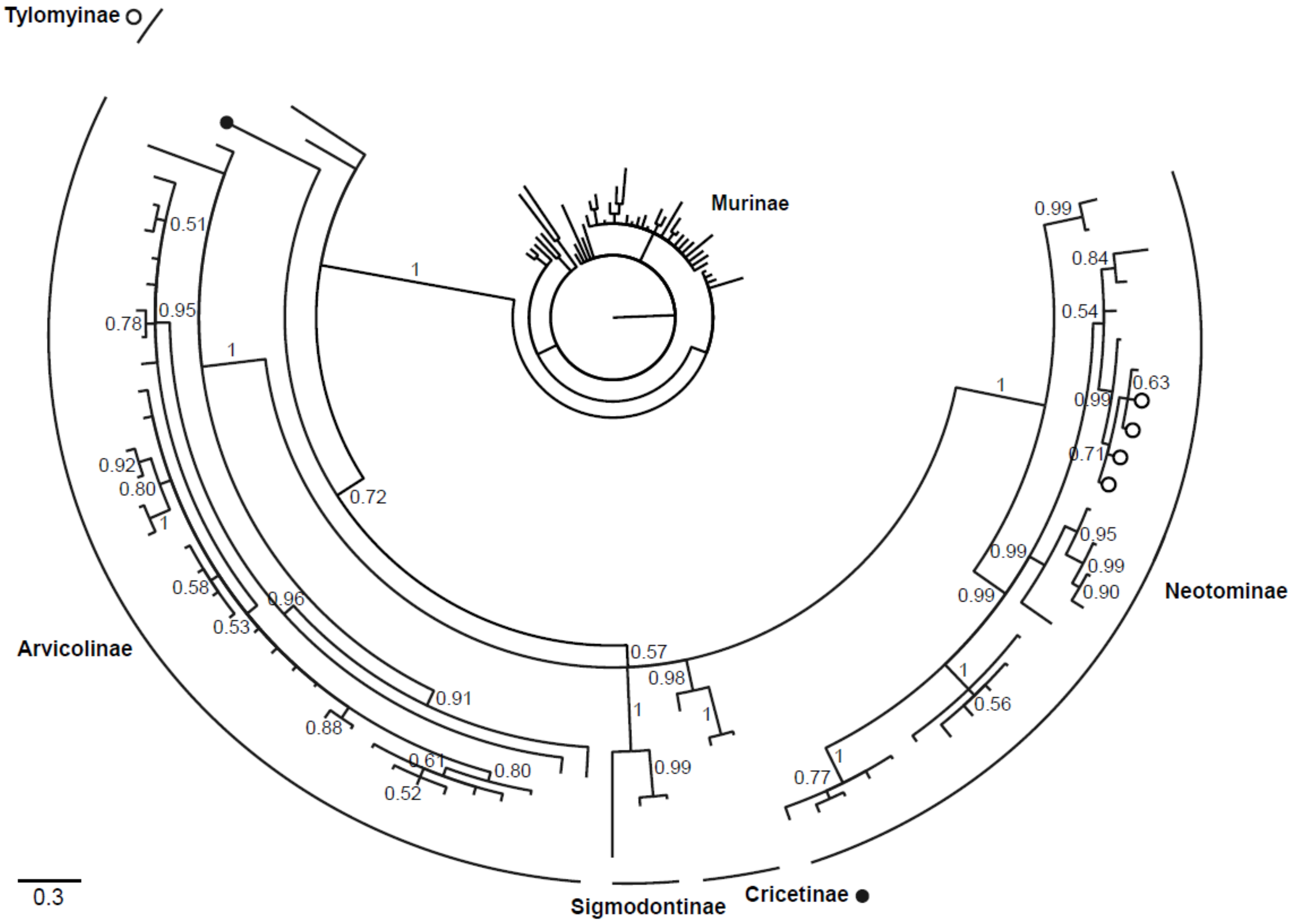

Figure S3 Amino acid sequence logos showing patterns of conservation and variation in the putative sperm-binding region of ZP3, for different data sets: a) Cricetidae + Murinae; b) Cricetidae; c) Murinae; d) Arvicolinae; e) Cricetinae; f) Neotominae; g) Sigmodontinae; and h) Tylomyinae. The overall height of each stack indicates the sequence conservation at the position, and the height of symbols within a stack reflects the relative frequency of the corresponding amino acid at each position, with the most frequent ('consensus') amino acid being on top of the stack. The amino acids are represented by their one-letter codes.

a)

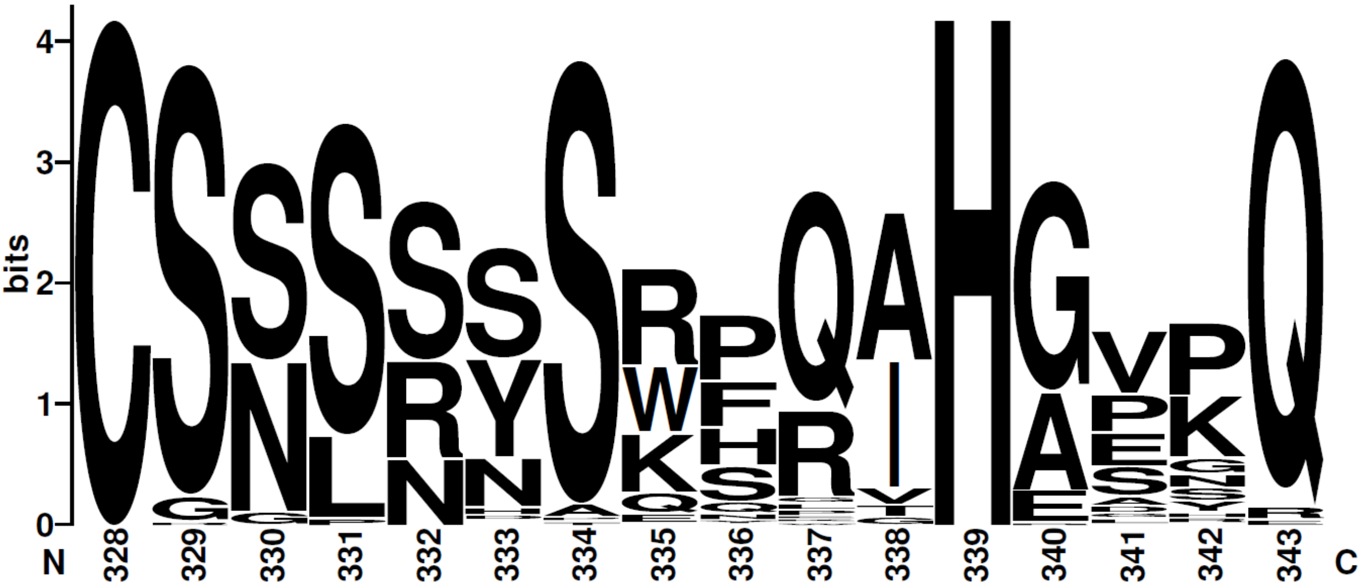

b)

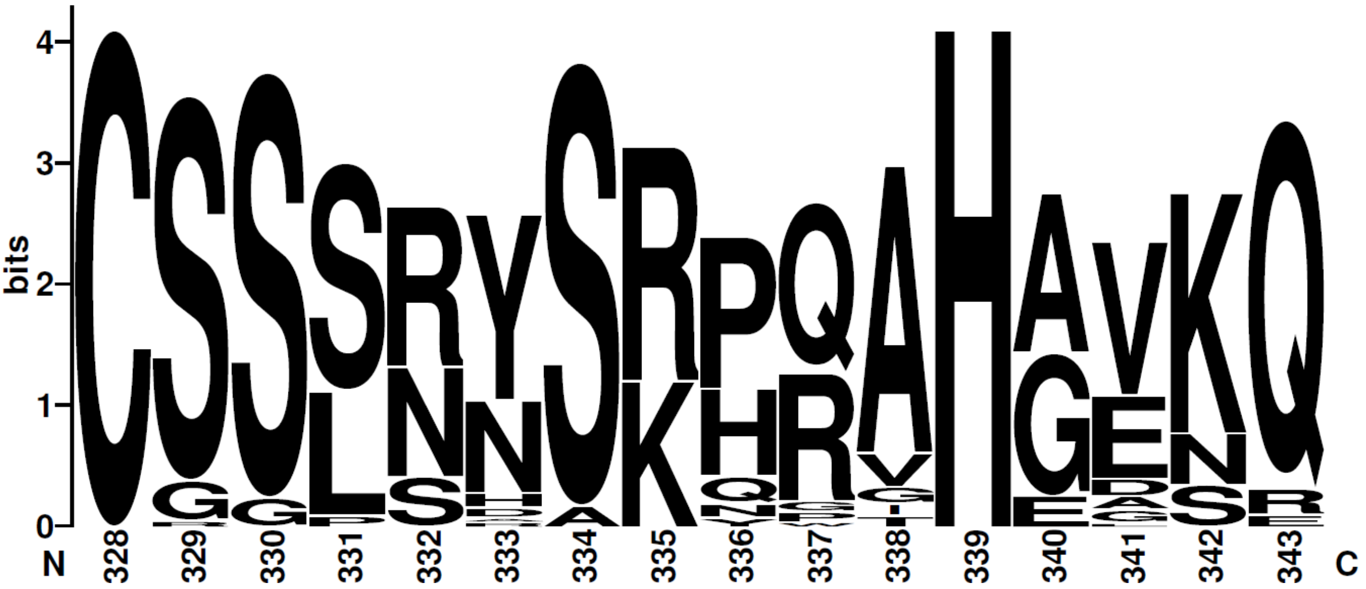

c)

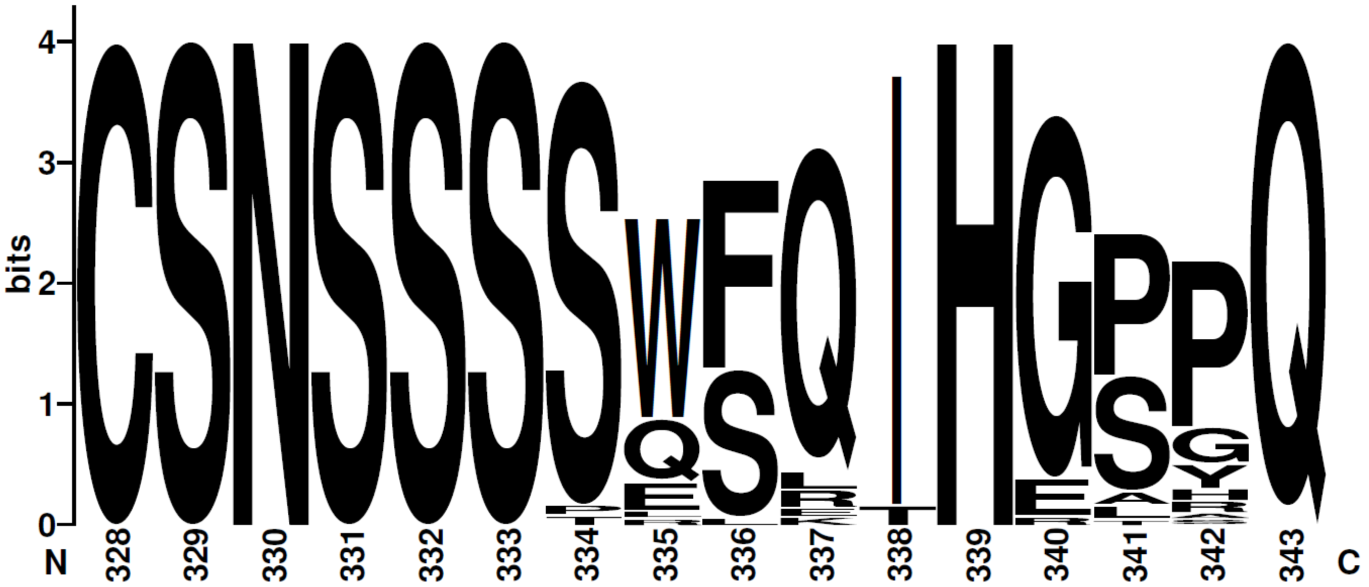
bits
0
1
2
3
4
N
328 329 330 331 332 333 334 335 336 337 338 339 340 341 342 343
C

d)

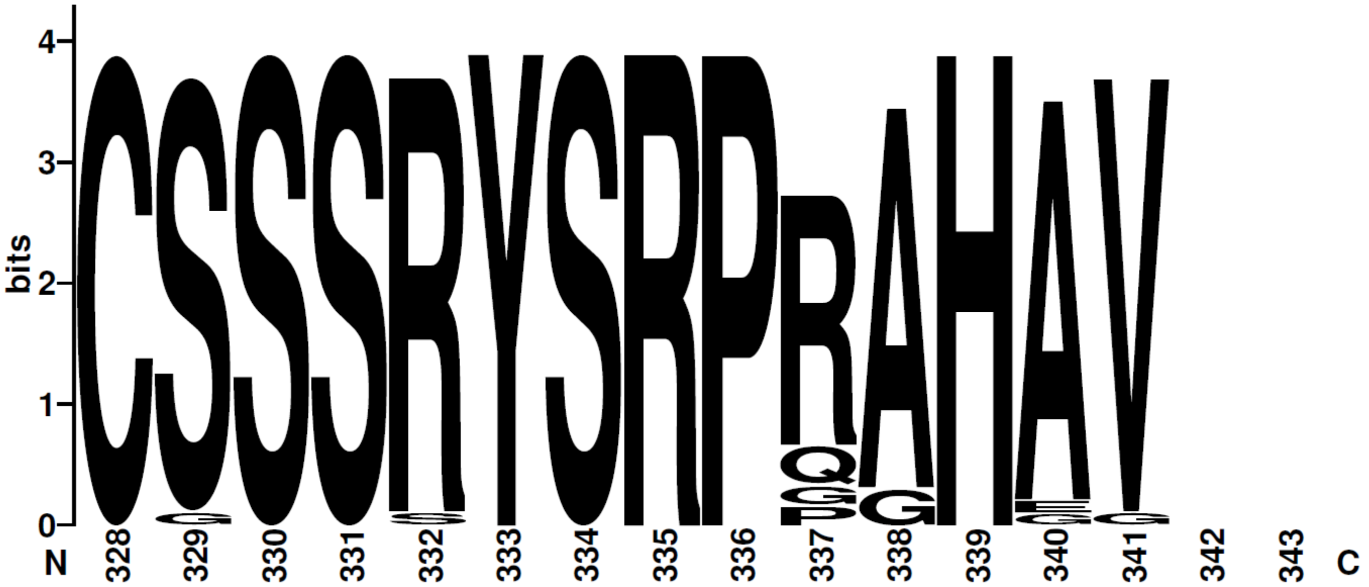
bits
0
1
2
3
4
N
328 329 330 331 332 333 334 335 336 337 338 339 340 341 342 343
C

e)

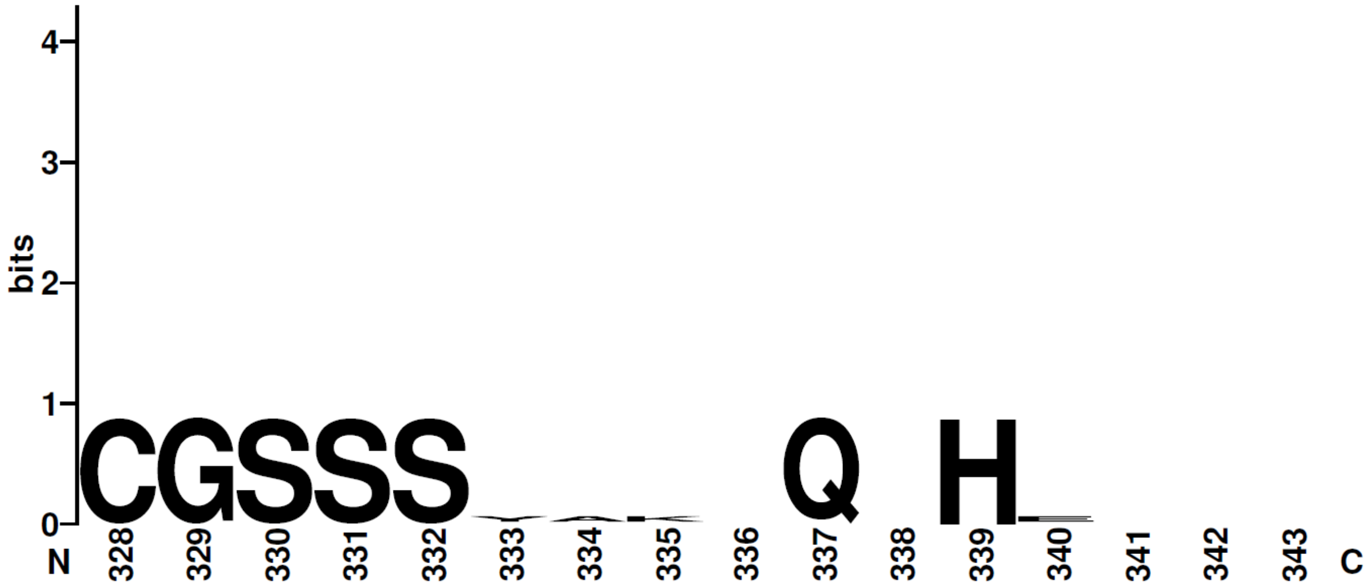
bits
0
1
2
3
4
N
328 329 330 331 332 333 334 335 336 337 338 339 340 341 342 343
C

f)

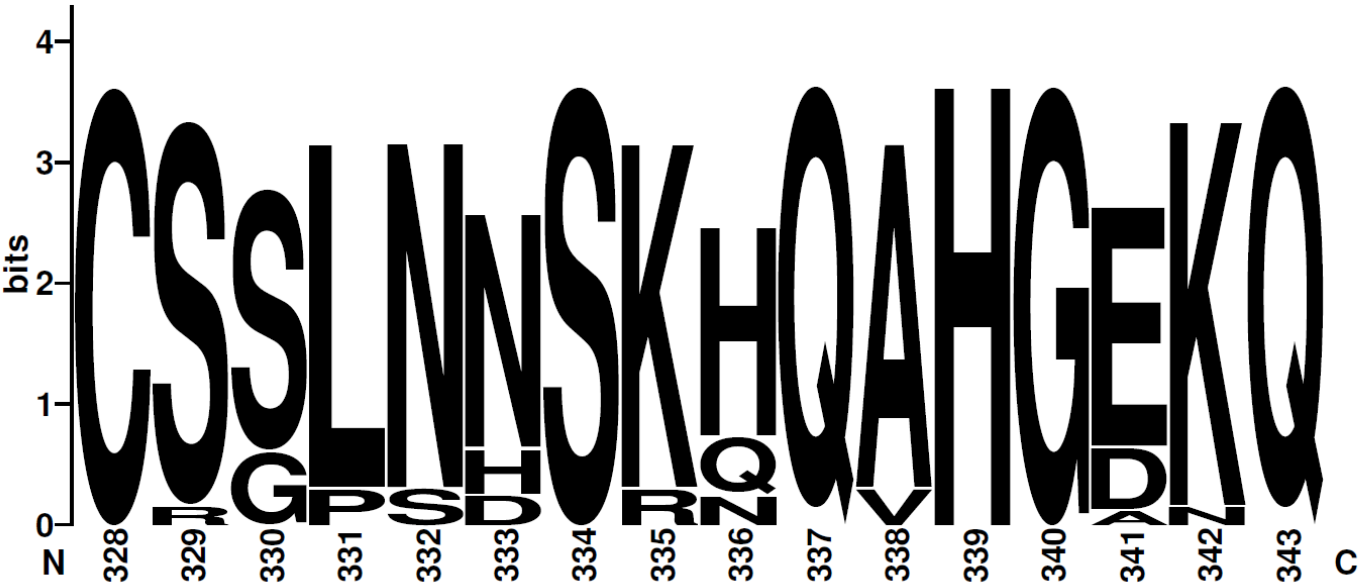
bits
0
1
2
3
4
N
328
329
330
331
332
333
334
335
336
337
338
339
340
341
342
343
C

g)

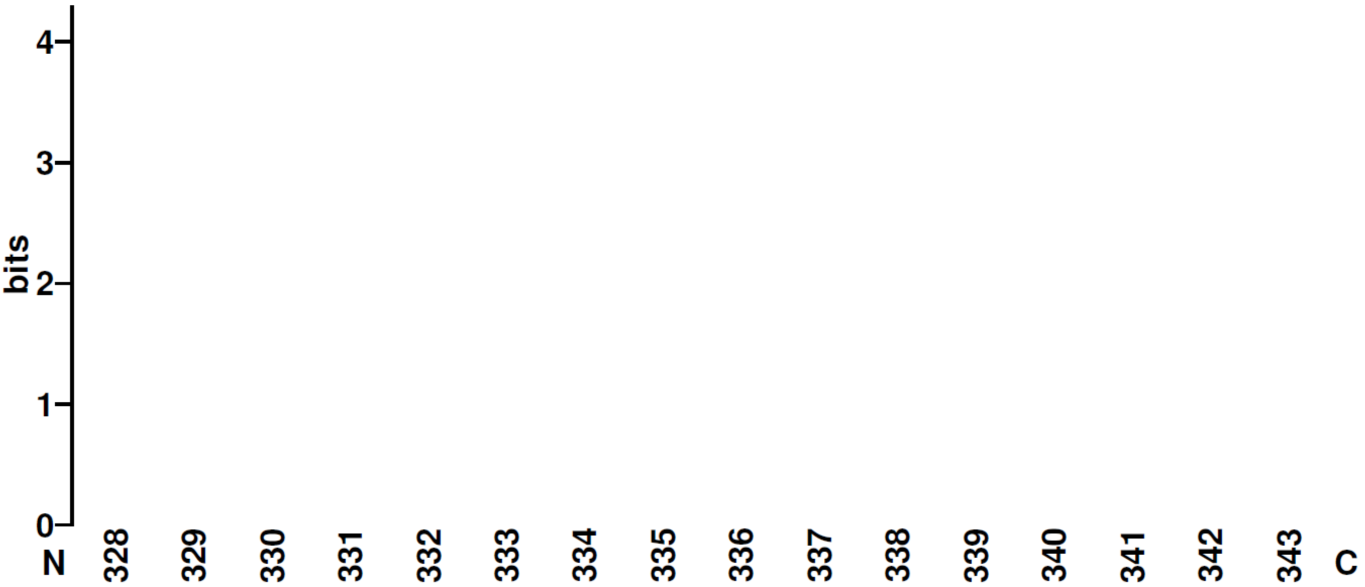
bits
0
1
2
3
4
N
328
329
330
331
332
333
334
335
336
337
338
339
340
341
342
343
C

h)

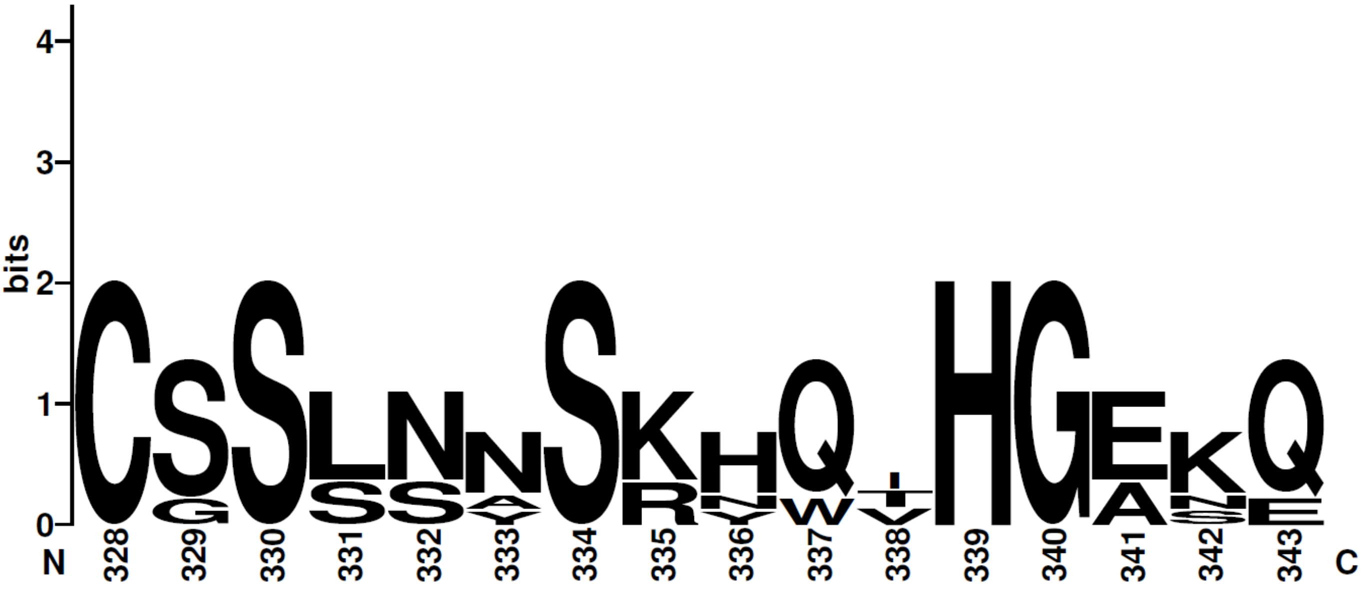
bits
0
1
2
3
4
N
328
329
330
331
332
333
334
335
336
337
338
339
340
341
342
343
C

Table S1 List of species examined in this study. This table provides information on the subfamily and family to which the species belong, whether the ZP3 sequence fragment was obtained in this study from specimens (in which case the acronym of the institution that provided the sample, and its voucher number if available, is given) or retrieved from Genbank, number of specimens sequenced per species (N), the accession numbers of the GenBank sequences, and the DNA and amino acid haplotypes found in each species. MTTU = Museum of Texas Tech University, USA; MNCN = Museo Nacional de Ciencias Naturales, Spain; CMPG = Institute of Ecology and Evolution, University of Bern, Switzerland; IZ-MLU = Institute of Zoology, Martin-Luther University, Germany; MUHNAC = Museu Nacional de História Natural e da Ciência, Portugal.

Species	Subfamily, Family	Source	N	GenBank accession number	DNA haplotype	Amino acid haplotype
Arvicola sapidus	Arvicolinae, Cricetidae	MUHNAC	2	MT226293	H1	A1
Chionomys nivalis		CMPG	2	MT226294	H2	A2
Lagurus lagurus		GenBank	-	AF515621	H3	A3
Lasiopodomys brandti		CMPG	1	MT226295	H4	A2
Microtus agrestis		MNCN (ES-20909)	1	MT226296<br>MT226297	H5<br>H6	A2
Microtus arvalis		CMPG	2	MT226298<br>MT226299<br>MT226300<br>MT226301<br>MT226302<br>MT226303<br>MT226304<br>MT226305	H7<br>H8<br>H9<br>H10<br>H11<br>H12<br>H13<br>H14	A2<br>A4
Microtus cabrerae		MUHNAC	3	MT226306<br>MT226307	H15<br>H16	A5
Microtus californicus		CMPG	1	MT226308	H17	A2
Microtus duodecimcostatus		MUHNAC	25	MT226298<br>MT226301<br>MT226309	H7<br>H10<br>H18	A2<br>A6
Microtus felteni		CMPG	1	MT226298<br>MT226305	H7<br>H14	A2
Microtus kikuchii		CMPG	1	MT226310<br>MT226311	H19<br>H20	A4
Microtus lusitanicus		MUHNAC	29	MT226298<br>MT226312	H7<br>H21	A2
Microtus montanus		CMPG	2	MT226313	H22	A7
Microtus montebelli		CMPG	1	MT226313	H22	A7
Microtus multiplex		CMPG	2	MT226314<br>MT226315	H23<br>H24	A8
Microtus ochrogaster		CMPG	2	MT226316<br>MT226317	H25<br>H26	A2

				MT226318	H27	
Microtus oeconomus		CMPG	1	MT226319	H28	A9
Microtus richardsoni		CMPG	1	MT226310<br>MT226311	H19<br>H20	A4
Microtus rossiaemeridionalis		CMPG	2	MT226298<br>MT226299	H7<br>H8	A2<br>A4
Microtus schelkovnikovi		CMPG	1	MT226295	H4	A2
Microtus socialis		CMPG	2	MT226320<br>MT226321<br>MT226322<br>MT226323<br>MT226324<br>MT226325	H29<br>H30<br>H31<br>H32<br>H33<br>H34	A2<br>A8<br>A10<br>A11<br>A12<br>A13
Microtus subterraneus		CMPG	1	MT226295	H4	A2
Microtus tatricus		CMPG	2	MT226298	H7	A2
Microtus thomasi		CMPG	2	MT226298	H7	A2
Myodes glareolus		MNCN (MNCN-144800/ MNCN-144801)	2	MT226326	H35	A14
Mesocricetus auratus	Cricetinae, Cricetidae	IZ-MLU	2	MT226280	H36	A15
Phodopus campbelli			1	MT226281	H37	A16
Phodopus roborovskii			3	MT226282	H38	A17
Phodopus sungorus			3	MT226283	H39	A16
Nyctomys sumichrasti	Tylomyinae, Cricetidae	MTTU (TK 19590 / TK 113595)	2	MT226284<br>MT226285<br>MT226286<br>MT226287<br>MT226288	H40<br>H41<br>H42<br>H43<br>H44	A18<br>A19<br>A20<br>A36<br>A37
Tylomys watsoni		MTTU (TK 136061)	1	MT226289	H45	A21
Oryzomys couesi	Sigmodontinae, Cricetidae	MTTU (TK 92437)	1	MT226290<br>MT226291	H46<br>H47	A22
Sigmodon arizonae		MTTU (TK 112658)	1	MT226292	H48	A23
Onychomys torridus	Neotominae, Cricetidae	GenBank	-	DQ668293+ DQ668343<br>DQ668292+ DQ668342	H49<br>H50	A24<br>A25
Peromyscus aztecus				DQ668245+ DQ668332	H51	A26
Peromyscus boylii				DQ668250+ DQ668323	H52	A27
Peromyscus californicus				DQ668253+ DQ668341	H53	A28

Peromyscus crinitus				DQ668255+ DQ668306	H54	A29
Peromyscus difficilis				DQ668259+ DQ668313	H55 H56 H57 H58	A30 A31
Peromyscus eremicus				DQ668262+ DQ668310	H59	A32
Peromyscus eva				DQ668263+ DQ668337	H60	A32
Peromyscus fraterculus				DQ668264+ DQ668338	H61	A32
Peromyscus gossypinus				DQ668267+ DQ668319	H62	A33
Peromyscus gratus				EU568656	H63	A34
Peromyscus leucopus				DQ668270+ DQ668316	H62 H64	A33
Peromyscus maniculatus				DQ668276+ DQ668299	H65 H66	A33
Peromyscus melanophrys				DQ668279+ DQ668336	H67	A35
Peromyscus mexicanus				DQ668283+ DQ668330	H68 H69	A36 A37
Peromyscus polionotus				EU489722	H70	A38
Peromyscus truei				EU568744	H71	A39
Aethomys ineptus	Murinae, Muridae	GenBank	-	EU004038	H72	A40
Anisomys imitator				EF364448	H73	A41
Apodemus chevrieri				EU004040	H74	A42
Bandicota indica				EU004041	H75	A43
Bunomys andrewsi				EU004042	H76	A44
Chiruromys vates				EF364449	H77	A45
Coccymys ruemmleri				EF364450	H78	A46
Conilurus penicillatus				EF364451	H79	A47
Crossomys moncktoni				EF364452	H80	A48
Dasymys incomtus				EU004043	H81	A49
Hydromys chrysogaster				EF364453	H82	A48
Hylomyscus alleni				AY057789	H83	A50
Hyomys goliath				EF364454	H84	A41
Leggadina forresti				EF364455	H85	A48
Lemniscomys griselda				EU004044	H86	A51
Leopoldamys sabanus				EU004046	H87	A52
Leporillus conditor				EF364457	H88	A53
Leptomys elegans				EF364458	H89	A48
Lorentzimys nouhuysi				EF364459	H90	S54
Macruromys major				EF364460	H91	A55

Mallomys aroensis				EF364461	H78	A46
Mammelomys rattoides				EF364464	H92	A56
Mastacomys fuscus				EF364465	H93	A57
Mastomys hildebrandtii				AY057790	H94	A58
Maxomys bartelsii				EU004047	H95	A59
Melomys cervinipes				EF364469	H96	A60
Mesembriomys gouldii				EF364472	H97	A61
Micaelamys namaquensis				EU004039	H98	A62
Mus musculus				M20026	H111	A72
Niviventer fulvescens				EU004049	H100	A63
Notomys alexis				EF364474	H99	A57
Parahydromys asper				EF364479	H101	A48
Paramelomys rubex				EF364482	H102	A64
Paruromys dominator				EU004050	H103	A43
Pogonomys macrourus				EF364484	H104	A65
Pseudohydromys ellermani				EF364466	H82	A48
Pseudomys laborifex				EF364499	H105	A66
Rhabdomys pumilio				EU004064	H107	A68
Rattus rattus				Y10823	H106	A67
Solomys salebrosus				EF364507	H108	A69
Uromys anak				EF364508	H109	A70
Xeromys myoides				EF364510	H82	A48
Zyzomys pedunculatus				EF364514	H110	A71

Table S2 Detailed results of selection tests. For the PAML models, the log-likelihood (*l*) of each model is given, as well as the position of positively selected codons (where ω > 1) identified by the Naive Empirical Bayes (NEB) and Bayes Empirical Bayes (BEB) methods. For the HyPhy tests, both positively and negatively selected sites are shown. Sites inferred to be under selection by the different site models in PAML or by the various site tests in HyPhy are underlined, while sites inferred to be under selection by the different site tests in both PAML and HyPhy are double underlined. *p0* = proportion of sites where ω < 1 (ω0), *p1* = proportion of sites where ω = 1 (ω1), *p2* = proportion of sites where ω > 1 (ω2), p/q = parameters of the beta distribution, [n] = positively selected site identified by NEB, [b] = positively selected site identified by BEB, * = probability > 95%, ** = probability > 99%, N.A. = not applicable.

Dataset	Software	Model or test	Parameters	κ (ts/tv)	Log-likelihood (*l*)	Positively selected sites	Negatively selected sites
Cricetidae + Murinae	PAML	M0:<br>one ratio	ω = 0.45307	4.27867	-2823.044887	N.A.	N.A.
		M1a:<br>nearly neutral	*p0* = 0.69290<br>ω0 = 0.10473<br>*p1* = 0.30710<br>ω1 = 1.00000	4.19730	-2715.812590	N.A.	N.A.
		M2a:<br>positive selection	*p0* = 0.64858<br>ω0 = 0.10658<br>*p1* = 0.25716<br>ω1 = 1.00000<br>*p2* = 0.09426<br>ω2 = 2.74189	4.77821	-2697.066353	311[nb]<br>325[nb]**<br>336[nb]*<br>337[nb]**<br>341[nb]**<br>342[nb]**<br>346[nb]	N.A.
		M3:<br>discrete	*p0* = 0.37891<br>ω0 = 0.00000<br>*p1* = 0.46362<br>ω1 = 0.39586<br>*p2* = 0.15747<br>ω2 = 2.05395	4.56989	-2686.221770	311[n]**<br>324[n]**<br>325[n]**<br>333[n]<br>335[n]**<br>336[n]**<br>337[n]**<br>338[n]*<br>341[n]**<br>342[n]**<br>346[n]**<br>347[n]	N.A.
		M7:<br>beta	p = 0.22452<br>q = 0.42241	4.07948	-2708.700999	N.A.	N.A.
		M8:<br>beta and ω > 1	*p0* = 0.87831<br>p = 0.32736<br>q = 0.92237<br>*p1* = 0.12169<br>ω = 2.27682	4.61074	-2687.502429	311[nb]<br>324[nb]<br>325[nb]**<br>335[nb]<br>336[nb]**<br>337[nb]**<br>341[nb]**<br>342[nb]**<br>346[nb]	N.A.
		M8a:<br>beta and ω = 1	*p0* = 0.79168<br>p = 0.46594<br>q = 2.64845	4.05849	-2705.263257	N.A.	N.A.

			p1 = 0.20832<br>ω = 1.00000				
		MA1,<br>foreground<br>branch<br>=<br>Murinae	N.A.	4.383	-1631.463527	315[bn]<br>322[bn]	N.A.
		MA1,<br>foreground<br>branch<br>=<br>Cricetidae	N.A.	4.082	-1626.069192	287[bn]<br>307[bn]*/**<br>311[bn]*	N.A.
		MA1,<br>foreground<br>branch<br>=<br>Arvicolinae	N.A.	4.226	-1628.893840	287[bn]**	N.A.
		MA1,<br>foreground<br>branch<br>=<br>Cricetinae	N.A.	4.242	-1631.709580	296[b]<br>309[b]	N.A.
		MA1,<br>foreground<br>branch<br>=<br>Neotominae	N.A.	4.237	-1632.173401	None	N.A.
		MA1,<br>foreground<br>branch<br>=<br>Sigmodontinae	N.A.	4.231	-1632.171253	None	N.A.
		MA1,<br>foreground<br>branch<br>=<br>Tylomyinae	N.A.	4.162	-1632.052340	307[b]<br>311[bn]	N.A.
	HyPhy	SLAC	N.A.	N.A.	N.A.	337*	287*<br>288**<br>289**<br>294**<br>295**<br>301**<br>302**<br>307*<br>312*<br>315*<br>323**<br>326**<br>328**<br>329*<br>334**<br>339**<br>354**
		FEL	N.A.	N.A.	N.A.	317*<br>336*<br>337**	280*<br>283*<br>287**<br>288*<br>289**<br>290*<br>294**<br>295**<br>296*<br>301**<br>302**<br>307**

							312**<br>315**<br>323**<br>326**<br>328**<br>334**<br>339**<br>354**
		MEME	N.A.	N.A.	N.A.	336*<br>337*	N.A.
		FUBAR	N.A.	N.A.	N.A.	336**<br>337**<br>342**	287**<br>289**<br>294**<br>295*<br>301*<br>302**<br>307*<br>312**<br>315*<br>323*<br>326**<br>328*<br>334**<br>339**<br>354**
Cricetidae	PAML	M0:<br>one ratio	ω = 0.40848	4.11044	-1840.140915	N.A.	N.A.
		M1a:<br>nearly neutral	*p0* = 0.58454<br>ω0 = 0.02766<br>*p1* = 0.41546<br>ω1 = 1.00000	4.21389	-1772.708134	N.A.	N.A.
		M2a:<br>positive selection	*p0* = 0.58109<br>ω0 = 0.02853<br>*p1* = 0.35888<br>ω1 = 1.00000<br>*p2* = 0.06003<br>ω2 = 2.11546	4.31392	-1772.345302	333[nb]<br>337[nb]	N.A.
		M3:<br>discrete	*p0* = 0.52941<br>ω0 = 0.01315<br>*p1* = 0.25547<br>ω1 = 0.55291<br>*p2* = 0.21512<br>ω2 = 1.48408	4.29966	-1770.261410	296[n]<br>297[n]<br>308[n]<br>321[n]<br>324[n]<br>325[n]*<br>333[n]**<br>336[n]<br>337[n]**<br>338[n]*<br>341[n]<br>342[n]<br>344[n]<br>346[n]<br>347[n]	N.A.
		M7:<br>beta	p = 0.09348<br>q = 0.14198	4.11249	-1772.441428	N.A.	N.A.
		M8:<br>beta and ω > 1	*p0* = 0.88218<br>p = 0.12025<br>q = 0.27748<br>*p1* = 0.11782<br>ω = 1.70965	4.30832	-1770.207833	293[b]<br>296[b]<br>297[b]<br>308[b]<br>317[b]<br>321[b]<br>324[b]<br>325[nb]<br>329[b]<br>333[nb]<br>336[nb]<br>337[nb]*	N.A.

						338[nb]<br>341[nb]<br>342[nb]<br>344[b]<br>346[nb]<br>347[b]	
		M8a:<br>beta and ω = 1	*p0* = 0.64093<br>p = 0.18762<br>q = 2.45890<br>*p1* = 0.35907<br>ω = 1.00000	4.13917	-1771.953080	N.A.	N.A.
	HyPhy	SLAC	N.A.	N.A.	N.A.	337*	287*<br>289**<br>298*<br>301**<br>312*<br>323*<br>326**<br>330*<br>334**<br>339**<br>354**
		FEL	N.A.	N.A.	N.A.	317*<br>337*	287**<br>289**<br>294*<br>295*<br>298*<br>301**<br>307*<br>312*<br>314*<br>315*<br>318*<br>323**<br>326**<br>328*<br>334*<br>339**<br>354**
		MEME	N.A.	N.A.	N.A.	297*<br>337*	N.A.
		FUBAR	N.A.	N.A.	N.A.	337**	287**<br>289*<br>301*<br>312*<br>323*<br>326**<br>334**<br>339**<br>354**
Murinae	PAML	M0:<br>one ratio	ω = 0.40348	3.31445	-1135.898833	N.A.	N.A.
		M1a:<br>nearly neutral	*p0* = 0.76934<br>ω0 = 0.07569<br>*p1* = 0.23066<br>ω1 = 1.00000	3.06737	-1095.799952	N.A.	N.A.
		M2a:<br>positive selection	*p0* = 0.77312<br>ω0 = 0.10194<br>*p1* = 0.13762<br>ω1 = 1.00000<br>*p2* = 0.08926<br>ω2 = 3.13645	3.69926	-1087.463200	311[nb]<br>325[nb]**<br>335[nb]<br>337[nb]<br>342[nb]**<br>346[nb]	N.A.
		M3:<br>discrete	*p0* = 0.48367<br>ω0 = 0.00000<br>*p1* = 0.39562<br>ω1 = 0.36774	3.70074	-1084.433658	279[n]<br>311[n]**<br>324[n]<br>325[n]**	N.A.

			p2 = 0.12071<br>ω2 = 2.68516			335^{n}**<br>337^{n}*<br>341^{n}<br>342^{n}**<br>346^{n}	
		M7:<br>beta	p = 0.12345<br>q = 0.29286	3.11705	-1095.892379	N.A.	N.A.
		M8:<br>beta and ω > 1	*p0* = 0.89108<br>p = 0.30707<br>q = 1.32853<br>*p1* = 0.10892<br>ω = 2.81543	3.69150	-1085.310331	279^{nb}<br>311^{nb}**/*<br>324^{n}<br>325^{nb}**<br>335^{nb}*<br>337^{nb}<br>341^{nb}<br>342^{nb}**<br>346^{nb}	N.A.
		M8a:<br>beta and ω = 1	*p0* = 0.80021<br>p = 0.41353<br>q = 3.92123<br>*p1* = 0.19979<br>ω = 1.00000	3.04708	-1094.453451	N.A.	N.A.
	HyPhy	SLAC	N.A.	N.A.	N.A.	None	289*<br>294*<br>295*<br>296*<br>302**<br>306*<br>327*<br>339**
		FEL	N.A.	N.A.	N.A.	342*	288**<br>289**<br>294*<br>295*<br>296**<br>302**<br>306**<br>307*<br>327*<br>339**
		MEME	N.A.	N.A.	N.A.	316*<br>342*	N.A.
		FUBAR	N.A.	N.A.	N.A.	355**<br>342**	288*<br>289*<br>294*<br>295*<br>296*<br>302**<br>306*<br>339**
Microtus	PAML	M0:<br>one ratio	ω = 0.15381	5.07385	-706.765944	N.A.	N.A.
		M1a:<br>nearly neutral	*p0* = 0.90975<br>ω0 = 0.00997<br>*p1* = 0.09025<br>ω1 = 1.00000	4.70089	-680.735490	N.A.	N.A.
		M2a:<br>positive selection	*p0* = 0.91830<br>ω0 = 0.01498<br>*p1* = 0.05719<br>ω1 = 1.00000<br>*p2* = 0.02452<br>ω2 = 4.30461	5.07955	-676.424362	297^{nb}<br>337^{nb}**	N.A.
		M3:<br>discrete	*p0* = 0.91526<br>ω0 = 0.01413<br>*p1* = 0.05823<br>ω1 = 0.92183<br>*p2* = 0.02652	5.03878	-676.613865	297^{n}<br>337^{n}**	N.A.

			ω2 = 4.03886				
		M7:<br>beta	p = 0.01066<br>q = 0.07279	4.71871	-680.839279	N.A.	N.A.
		M8:<br>beta and ω > 1	*p0* = 0.96968<br>p = 0.03462<br>q = 0.51150<br>*p1* = 0.03032<br>ω = 3.80463	5.01679	-676.664233	297[nb]<br>337[nb]**	N.A.
		M8a:<br>beta and ω = 1	*p0* = 0.90974<br>p = 1.03990<br>q = 99.00000<br>*p1* = 0.09026<br>ω = 1.00000	4.69391	-680.780313	N.A.	N.A.
	HyPhy	SLAC	N.A.	N.A.	N.A.	337*	291**<br>318**<br>326*<br>334*<br>339*
		FEL	N.A.	N.A.	N.A.	337*	291**<br>303*<br>314*<br>315*<br>318**<br>326*<br>333*<br>334*<br>339*
		MEME	N.A.	N.A.	N.A.	337*	N.A.
		FUBAR	N.A.	N.A.	N.A.	337**	291**<br>318**<br>326**<br>334**<br>339*
Peromyscus	PAML	M0:<br>one ratio	ω = 0.35231	3.83664	-735.567226	N.A.	N.A.
		M1a:<br>nearly neutral	*p0* = 0.61897<br>ω0 = 0.00000<br>*p1* = 0.38103<br>ω1 = 1.00000	4.07239	-728.070941	N.A.	N.A.
		M2a:<br>positive selection	*p0* = 0.61897<br>ω0 = 0.00000<br>*p1* = 0.28498<br>ω1 = 1.00000<br>*p2* = 0.09605<br>ω2 = 1.00000	4.07238	-728.070941	333[b]	N.A.
		M3:<br>discrete	*p0* = 0.34058<br>ω0 = 0.00000<br>*p1* = 0.27098<br>ω1 = 0.00000<br>*p2* = 0.38844<br>ω2 = 0.94086	4.02303	-728.050079	None	N.A.
		M7:<br>beta	p = 0.01629<br>q = 0.02858	4.04372	-728.072655	N.A.	N.A.
		M8:<br>beta and ω > 1	*p0* = 0.87803<br>p = 0.01356<br>q = 0.03717<br>*p1* = 0.12197<br>ω = 1.00000	4.04761	-728.060149	333[n]<br>344[n]<br>346[n]	N.A.
		M8a:<br>beta and ω = 1	*p0* = 0.61897<br>p = 0.00500<br>q = 74.14239<br>*p1* = 0.38103<br>ω = 1.00000	4.07239	-728.070936	N.A.	N.A.
	HyPhy	SLAC	N.A.	N.A.	N.A.	None	287*<br>289*<br>298*

							323**<br>328*<br>354*
		FEL	N.A.	N.A.	N.A.	None	287**<br>289**<br>298**<br>323**<br>326*<br>328**<br>354**
		MEME	N.A.	N.A.	N.A.	None	N.A.
		FUBAR	N.A.	N.A.	N.A.	None	287**<br>289*<br>298*<br>323**<br>328*<br>342*<br>354*
